# Biomechanical Modeling and Analysis of the Lower-Limb Musculoskeletal System for Hemiplegia: A Pilot Study

**DOI:** 10.3390/s26113353

**Published:** 2026-05-25

**Authors:** Kexiang Li, Ye Sun, Chuang Li, Tongzan Guo, Hui Li

**Affiliations:** 1 College of Mechanical and Material Engineering, North China University of Technology, Beijing 100144, China; likex_2023@ncut.edu.cn (K.L.); 2024312070127@mail.ncut.edu.cn (Y.S.); 2024322100124@mail.ncut.edu.cn (C.L.); 2 College of Mechanical Engineering, Hebei University of Technology, Tianjin 300401, China; 3 College of Mechanical Engineering, University of Science and Technology Beijing, Beijing 100083, China

**Keywords:** sEMG, lower-limb musculoskeletal model, parameter optimization, hemiplegic patients, biomechanical analysis

## Abstract

Preliminary estimation of lower-limb motor function is important in rehabilitation research, especially for biomechanical assessment of post-stroke hemiplegic gait. However, subject-specific musculoskeletal modeling in this population is challenging because standard maximum voluntary contraction (MVC) testing is often unsafe or unreliable for normalizing surface electromyography (sEMG) signals. To address this limitation, a normalized correction coefficient was introduced for pathological sEMG preprocessing, and an improved Hill-type muscle model (iHMM) was established to account for submaximal activation conditions. By combining inverse dynamics, static optimization, and a subject-specific lower-limb dynamic model, the proposed framework was used to estimate musculotendon force, knee joint torque, knee joint kinematics, and shank center-of-mass trajectory. In a preliminary validation involving six hemiplegic subjects, the predicted knee joint torques showed moderate to good agreement with the reference results, with correlation coefficients ranging from 0.724 to 0.807 and RMSE values ranging from 3.872 to 7.814 Nm. These preliminary results support the feasibility of the proposed framework for subject-specific biomechanical analysis of the hemiplegic lower extremity and suggest its potential utility in individualized rehabilitation assessment.

## 1. Introduction

Lower limb motor dysfunction, a common sequela of stroke, severely impairs patients’ activities of daily living and quality of life [1]. Biomechanical analysis has emerged as a core tool in hemiplegic rehabilitation, boasting dual clinical values: on one hand, it provides clinicians with objective evaluation indicators for lower limb motor function, laying a scientific foundation for formulating personalized rehabilitation protocols; on the other hand, it synergizes with exoskeleton-assisted training systems to significantly enhance therapeutic outcomes [2,3,4]. However, most existing biomechanical studies focus on healthy populations, and systematic research targeting hemiplegic patients remains relatively scarce [5,6,7]. Compared with healthy individuals, hemiplegic stroke survivors have a more urgent demand for precise motor function assessment and individualized intervention strategies [8]. Therefore, developing accurate quantitative assessment methods for lower limb physiological dynamics and constructing efficient multidimensional biomechanical evaluation frameworks have become key research directions in rehabilitation engineering [9].

Pathological movement patterns in hemiplegic patients are closely associated with neuromuscular system impairments [10]. The precise quantification of muscle forces and joint torques during dynamic movements serves as the core foundation for optimizing personalized rehabilitation protocols for this clinical population. Although surface electromyography (sEMG) can accurately and non-invasively capture real-time muscle electrical activity, it cannot directly quantify key parameters such as internal muscle forces and joint torques. These intrinsic biomechanical indicators are crucial for deciphering the pathological mechanisms of abnormal gait but are difficult to directly measure in vivo through non-invasive means [11]. Thus, constructing an sEMG-driven musculoskeletal simulation model is of great significance for bridging the gap between measurable electrical signals and non-measurable intrinsic mechanical parameters.

In the research on human movement mechanisms, musculoskeletal system modeling is an effective research method that helps clarify how neural signals are transmitted to multiple muscle–tendon units spanning joints, ultimately driving joint movement [12]. In skeletal muscle models, the problem of muscle redundancy is inevitable—the number of muscles far exceeds the number of joint degrees of freedom they control [5,13], leading to an infinite number of solutions for muscle force distribution across joint degrees of freedom. Currently, muscle force prediction in skeletal muscle models mainly relies on optimization theory, which estimates muscle forces by minimizing a physiological cost objective function [14,15]. It is primarily divided into two methods: static optimization and dynamic optimization. Static optimization is based on the principle of inverse dynamics, estimating muscle forces from known motion states; dynamic optimization adopts the reverse solution logic [16,17].

Lower limb joint movement relies on the coordinated activation of multiple muscle units. sEMG signals can indirectly reflect an individual’s neural drive state and are easy to collect during human movement [18,19], thus being widely used to directly drive the simulation of upper and lower limb musculoskeletal models [12,20,21]. However, the core challenge in sEMG-driven modeling for hemiplegic patients lies in the signal normalization process: stroke patients are often accompanied by symptoms such as spasticity and muscle weakness, making reliable maximum voluntary contraction (MVC) tests both unsafe and nearly impossible to perform. Traditional MVC normalization methods can lead to significant deviations in muscle activation estimation. Data-driven methods, including machine learning approaches, have been increasingly explored for rehabilitation biomechanics and gait analysis [22]. Recent studies have shown that physics-informed deep learning can embed biomechanical constraints into data-driven musculoskeletal models for predicting muscle forces and joint kinematics from sEMG signals [23,24,25]. In addition, generative learning and data-balancing strategies have been used to improve gait classification under limited and imbalanced clinical datasets [26]. Nevertheless, robustly estimating internal muscle forces from surface sensor signals while maintaining clear physiological correspondence remains challenging. The mechanistic sEMG-driven model proposed in this study aims to provide a complementary approach by preserving explicit relationships among sEMG excitation, muscle activation, musculotendon mechanics, and joint-level biomechanical outputs. For physiological models, another key challenge is that an increase in the number of muscles in the model leads to a proportional increase in the number of parameters to be determined, thereby increasing the computational burden [27,28]. Nevertheless, physiological models still possess significant advantages: they can provide more comprehensive musculoskeletal biomechanical information, and more accurately replicate human movement patterns [29], and ultimately offer intuitive and feasible rehabilitation guidance for hemiplegic patients.

This study develops a subject-specific sEMG-driven musculoskeletal modeling framework to estimate knee joint torque and related biomechanical variables during hemiplegic gait. Lower-limb sEMG signals, marker trajectories, and ground reaction forces were collected and used to calibrate the musculoskeletal model. A bounded sEMG correction coefficient was applied to support activation estimation without relying on conventional MVC tests, and an improved Hill-type muscle model was employed to compute musculotendon forces. The framework was evaluated using held-out trials from the same subjects, and the results were interpreted as computational agreement rather than independent physiological validation, reflecting a preliminary feasibility assessment.

## 2. Method

**Figure 1:**
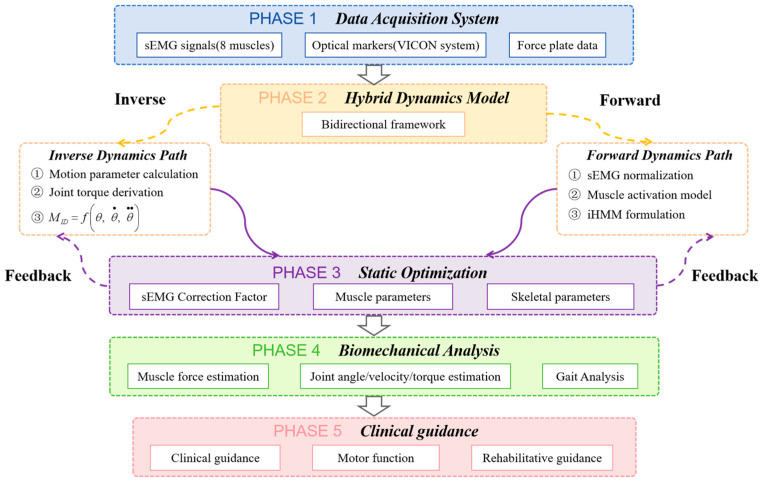
Overall conceptual framework of this study.

The conceptual framework of the proposed subject-specific sEMG-driven musculoskeletal modeling approach is shown in Figure 1. First, lower-limb sEMG signals, optical marker trajectories, and ground reaction forces were collected during overground walking. Next, a subject-specific musculoskeletal model was constructed using anthropometric scaling to capture individual lower-limb geometry. Because reliable MVC trials may be difficult for hemiplegic patients, a bounded sEMG correction coefficient was introduced to support MVC-free activation estimation. Activation estimates were then entered into an improved Hill-type muscle model to calculate musculotendon forces and joint-level biomechanical variables. Finally, the model outputs were evaluated using within-subject held-out trials and interpreted as preliminary subject-specific biomechanical estimates.

### 2.1. Skeletal Muscle Model

**Figure 2:**
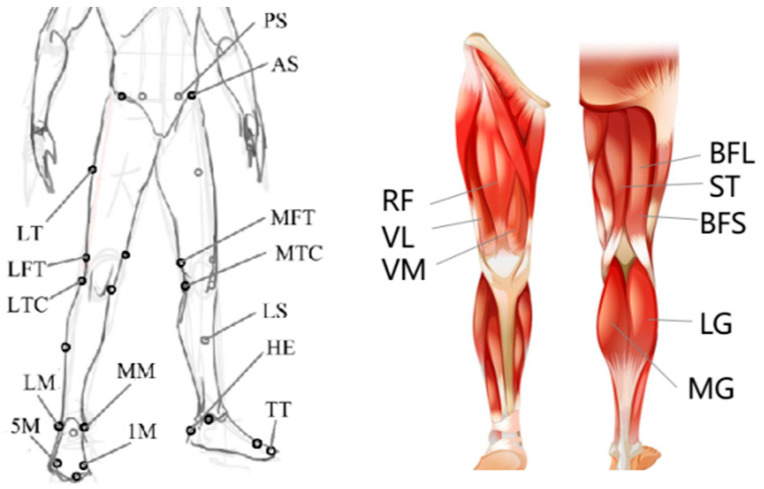
Human lower limb movement markers and related muscle position points.

To estimate lower-limb muscle forces and joint moments during gait, a sEMG-driven musculoskeletal framework was created by combining a subject-specific skeletal model with a lower-limb muscle model. The skeletal model represents the kinematic and dynamic structure of the lower extremities, whereas the muscle model specifies the musculotendon architecture necessary for force estimation. These two components form the mechanical foundation of the proposed system. Figure 2 shows the marker configuration utilized for lower-limb motion recording, as well as the positions of the key monitored muscles.

#### 2.1.1. Skeletal Model

A generic lower-limb musculoskeletal model was implemented in OpenSim as the baseline skeletal framework. Specifically, the popular gait2392_simbody model was chosen since it offers a physiologically significant depiction of the lower extremity and has been frequently utilized in biomechanical research pertaining to gait. With 92 musculotendon actuators and 23 degrees of freedom, the model can be used for subject-specific scaling, inverse kinematics, and inverse dynamics.

Anthropometric measurements and motion-capture data were used to scale the generic model to increase anatomical congruence with the testing subjects. The femur and tibia underwent manual refinement to better fit subject-specific geometry, and segment dimensions were modified based on the recorded marker trajectories. The normal OpenSim measurement-based process was used to scale the remaining body portions. Major lower-limb anatomical indicators were given comparatively large weights during scaling for the purpose of increasing the accuracy of hip, knee, and ankle kinematic reconstruction.

After scaling, inverse kinematics was performed to compute joint angles from the marker trajectories, and inverse dynamics was subsequently conducted to obtain the net joint moments during gait. To further minimize dynamic inconsistencies caused by measurement noise and modeling simplifications, a residual reduction analysis was performed. For further muscle-force measurement and model calibration, the generated subject-specific skeletal model served as the kinematic and dynamic reference.

#### 2.1.2. Muscle Model

The lower-limb musculature depicted in the scaled OpenSim model served as the foundation for the muscle component of the suggested framework. The monitored muscles associated with sagittal-plane gait mechanics included the lateral gastrocnemius (LG), medial gastrocnemius (MG), biceps femoris long head (BFL), biceps femoris short head (BFS), vastus medialis (VM), vastus lateralis (VL), rectus femoris (RF), and semitendinosus (ST). These muscles were chosen because they can be accurately quantified with sEMG and have a significant role in knee and ankle actuation during walking.

For each muscle, the associated musculotendon parameters included the optimal fiber length $l_{mo}$, tendon slack length $l_{ts}$, pennation angle φ, and maximal isometric force $F_{max}$. These factors directly affect the conversion of muscle activation into muscular force and establish the musculotendon unit’s ability to generate force. In order for the lower-limb muscle model to more accurately depict the biomechanical aspects of hemiplegic gait, certain musculotendon parameters were permitted to fluctuate during model creation, as generic parameter values cannot properly capture subject-specific neuromuscular characteristics. The generated muscle model served as the mechanical foundation for further sEMG-driven estimates of muscle force and joint moment [30].

### 2.2. Muscle Activation Model

Muscle activation is a key intermediate variable linking sEMG to muscle-force generation, and its preliminary estimation is essential for reliable muscle-mechanics modeling. A typical preprocessing method was used before activation estimates because raw sEMG data are prone to power-line interference, DC bias, motion aberrations, and low-frequency noise [31]. With the goal of eliminating low-frequency noise and baseline drift, the raw sEMG signals were first high-pass filtered using a fourth-order Butterworth filter with a cutoff frequency of 30 Hz. This cutoff frequency was selected to reduce motion-related low-frequency artifacts, electrode-skin movement, and baseline drift during overground hemiplegic gait recordings, while preserving a stable rectified envelope for subsequent muscle activation estimation. To create smooth linear envelopes, the filtered signals were first full-wave rectified and then low-pass filtered at 6 Hz using a fourth-order Butterworth filter. This process approximates the intrinsic low-pass filtering properties of muscle tissue while also suppressing measurement noise.

In conventional sEMG-driven models, signal normalization is typically performed using the maximum voluntary contraction (MVC) reference, where the maximum sEMG amplitude during MVC is taken as the 100% activation level. However, for hemiplegic subjects, reliable MVC measurements are often difficult to obtain because impaired motor control and reduced voluntary output prevent the achievement of true maximal contraction, while excessive exertion may also increase the risk of secondary injury. To address this limitation, a correction coefficient was introduced to replace the conventional MVC reference. Let $s\left(t\right)$ denote the preprocessed sEMG signal and $s_{max}$ its maximum value within the corresponding trial. The corrected normalized sEMG signal was defined as

(1) $$e\left(t\right)=\frac{s\left(t\right)}{s_{max}}\cdot c$$

where *c* is the sEMG normalization correction coefficient. In this study, *c* was defined as a bounded correction coefficient for MVC-free sEMG normalization, rather than as a direct clinical index of spasticity or voluntary activation capacity. The coefficient *c* was initialized at 0.50 and constrained within 0.10–1.00. This bounded range was used to allow MVC-free normalization while limiting excessive amplification of weak or noise-contaminated sEMG envelopes during parameter optimization. This formulation allows the normalization process to adapt to subject-specific neuromuscular characteristics while avoiding direct dependence on unreliable MVC measurements.

To describe the dynamic relationship between sEMG excitation and neural drive, a second-order recursive model was adopted to characterize neural excitation:

(2) $$\mu \left(t\right)=\alpha e\left(t-d\right)-\beta_{1}\mu \left(t-1\right)-\beta_{2}\mu \left(t-2\right)$$

where $\mu \left(t\right)$ denotes neural excitation, *d* is the electromechanical delay between sEMG excitation and estimated muscle activation, and *α*, $\beta_{1}$, and $\beta_{2}$ are model coefficients. The delay *d* was treated as an integer sample delay and searched within 10–40 samples; the selected delay was then converted to milliseconds according to the actual sEMG sampling frequency. The model coefficients were constrained during optimization to ensure numerical stability. An exponential transformation was used to generate the final muscle activation due to the nonlinear relationship between brain stimulation and muscle activation:

(3) $$a\left(t\right)=\frac{1-\exp\left(A\cdot \mu \left(t\right)\right)}{1-\exp\left(A\right)}$$

where $a\left(t\right)$ denotes muscle activation, and *A* is a nonlinear shape factor. This formulation constrains activation within a physiologically meaningful range while capturing the nonlinear recruitment characteristics of skeletal muscle under submaximal excitation.

### 2.3. Improved Hill Muscle Model (iHMM)

To estimate muscle force from the sEMG-derived activation, an improved Hill-type muscle model was adopted. This model provides a practical balance between physiological interpretability and computational efficiency, making it suitable for sEMG-driven musculoskeletal simulation in pathological gait analysis [32,33,34].

**Figure 3:**
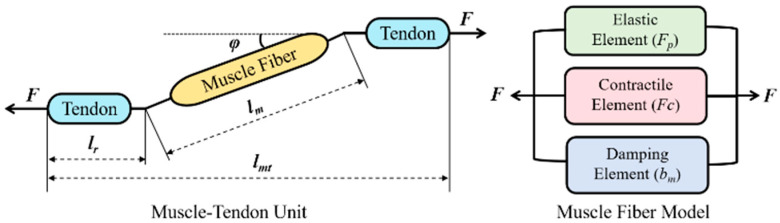
The improved Hill muscle model.

As illustrated in Figure 3, the musculotendon unit was represented by three components: a contractile element (CE), a parallel elastic element (PE), and a viscous damping element. Compared with the conventional Hill-type formulation, the introduction of the damping term allows the model to better characterize energy dissipation during muscle contraction under pathological gait conditions. Based on this structure, the force generated by the *i*-th muscle was expressed as

(4) $$F_{mt,i}\left(t\right)\;=F_{max,i}\left[a_{i}\left(t\right)\cdot f_{l}\left(l_{i}\left(t\right)\right)\cdot f_{v}\left(v_{m,i}\left(t\right)\right)+f_{p}\left(l_{m,i}\left(t\right)\right)+\beta \cdot v_{m,i}\left(t\right)\right]\cos\varphi_{i}\left(t\right)$$

where $F_{mt,i}\left(t\right)$ denotes the force generated by the *i*-th muscle-tendon unit at time *t*, $a_{i}\left(t\right)$ is the muscle activation obtained from Equation (3), $f_{l}\left(\cdot \right)$, $f_{v}\left(\cdot \right)$ and $f_{p}\left(\cdot \right)$ are the active force–length, force–velocity, and passive force–length relationships, respectively, $F_{max,i}$ is the maximal isometric force, $l_{m,i}\left(t\right)$ and $v_{m,i}\left(t\right)$ denote the muscle fiber length and contraction velocity, respectively, *β* is the damping coefficient, and $\varphi_{i}\left(t\right)$ is the pennation angle.

Because pennation angle varies dynamically with muscle fiber length, it was treated as a time-varying quantity rather than a fixed parameter. The pennation angle was calculated as

(5) $$\cos\varphi_{i}\left(t\right)=\sqrt{1-{\left(\frac{l_{opt,i}\cdot sin\varphi_{0,i}}{l_{m,i}\left(t\right)}\right)}^{2}}$$

where $\varphi_{0,i}$ is the pennation angle at the optimal fiber length; $l_{mo,i}$ is the optimal fiber length; and $l_{m,i}\left(t\right)$ is the instantaneous muscle fiber length of the *i*-th muscle at time *t*. The dynamic pennation-angle formulation was based on the commonly used geometric approximation in Hill-type musculotendon modeling [35]. Because hemiplegic muscles may exhibit atrophy, fibrosis, or altered architecture, this formulation was treated as a modeling approximation rather than as a direct subject-specific measurement of pennation behavior. The classical Hill-type model is typically formulated for a fully activated muscle. However, during daily locomotion, especially in hemiplegic gait, muscles rarely operate under maximal activation [36]. To improve force prediction under submaximal activation, an activation-dependent adjustment of the optimal fiber length was introduced:

(6) $$l_{mo,i}^{*}\left(t\right)=l_{mo,i}\left(\gamma \left(1-a_{i}\left(t\right)\right)+1\right)$$

where γ is the percentage change factor of the optimal fiber length, $l_{mo,i}$ is the baseline optimal fiber length under maximum activation, and $l_{mo,i}^{*}\left(t\right)$ is the time-varying optimal fiber length adjusted for the submaximal activation level $a_{i}\left(t\right)$. This modification allows the force–length relationship to vary with activation level and improves the model’s ability to represent muscle mechanics under non-maximal contraction.

Compared with a purely generic Hill-type formulation, the present model was improved in two aspects [37]. First, the activation input was derived from corrected sEMG signals rather than directly from conventional MVC-normalized sEMG, which is more suitable for hemiplegic subjects. Second, selected musculotendon parameters were adjusted in a subject-specific manner, allowing the model to better reflect the altered mechanical behavior of pathological muscle.

At each time instant, the estimated muscle forces were projected to the corresponding joint through muscle moment arms to obtain the model-estimated joint moment:

(7) $$M_{model}\left(t\right)\;=\sum_{i=1}^{n}r_{i}\left(t\right)\;F_{mt,i}\left(t\right)$$

where *n* is the number of muscles contributing to the target joint, $r_{i}\left(t\right)$ is the moment arm of the *i*-th muscle, and $F_{mt,i}\left(t\right)$ is the corresponding muscle force. The resulting joint moment served as the mechanical output of the proposed sEMG-driven model.

### 2.4. Model Calibration

Three main parts make up the suggested sEMG-driven musculoskeletal model: a lower-limb joint geometry model, an enhanced Hill-type muscle mechanics model, and a sEMG-based muscle activation model [38]. To increase consistency between model predictions and experimental gait data, a subject-specific calibration approach was established because generic parameter values are unable to properly capture the neuromuscular and biomechanical properties of hemiplegic participants.

**Figure 4:**
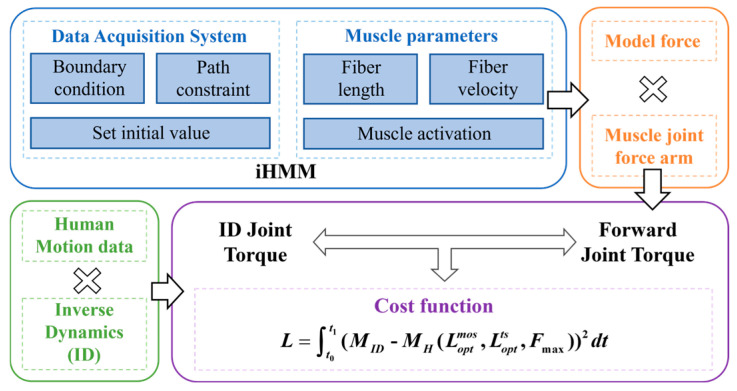
Parameter optimization flowchart.

The calibration variables included the sEMG normalization correction coefficient *c*, damping coefficient *B*, optimal fiber length scaling of $l_{mo}$, and tendon slack length scaling of $l_{ts}$. To reduce parameter equifinality between activation scaling and force-generating capacity, the maximal isometric force $F_{max}$ was fixed to the value obtained from the subject-specific scaled musculoskeletal model and was not optimized simultaneously with *c*. The remaining calibration variables were constrained within predefined bounded ranges to improve numerical stability and reduce non-physiological parameter compensation. The overall parameter optimization procedure is illustrated in Figure 4. These parameters were selected because they directly affect the mechanical output of the model and may vary substantially across hemiplegic subjects.

A constrained nonlinear optimization problem was used to formulate the calibration problem [39]. The total squared errors between the inverse-dynamics reference moments and the model-estimated joint moments over the gait cycle were used to establish the objective function:

(8) $$J=\sum_{t=1}^{N}{\left[M_{model}\left(t\right)-M_{ID}\left(t\right)\right]}^{2}$$

where $M_{model}\left(t\right)$ is the joint moment predicted by the sEMG-driven musculoskeletal model and obtained from the musculotendon forces according to Equation (7), $M_{ID}\left(t\right)$ is the corresponding inverse-dynamics joint moment, and *N* is the number of sampled time frames within the gait cycle. To ensure physiological plausibility and numerical stability, all calibration variables were constrained within predefined lower and upper bounds:

(9) $$p_{i}^{\min}\leq p_{i}\leq p_{i}^{\max},\;i=1,2,\ldots ,m$$

where $p_{i}$ denotes the *i*-th calibration parameter, and *m* is the total number of optimized parameters. The bounds were specified according to physiologically admissible parameter ranges and the corresponding generic-model settings, thereby preventing the optimization from converging to non-physiological solutions. In addition, muscle activation was constrained within the feasible range $0\leq a_{i}\left(t\right)\leq 1$.

### 2.5. Human Lower Limb Dynamics

After measuring the musculotendon forces, the related joint moments and lower-limb dynamic variables were calculated to characterize the mechanical behavior of gait. Because each musculotendon unit’s contribution to joint rotation is determined by its moment arm, the muscle moment arm was initially estimated using the virtual work principle [40]. Using the tendon displacement approach, the moment arm of the *k*-th degree of freedom was defined as

(10) $$r_{k}=\frac{\partial L\left(q\right)}{\partial q_{k}}$$

where $L\left(q\right)$ denotes the musculotendon length as a function of joint angle *q*, and $r_{k}$ is the corresponding muscle moment arm. In this study, a five-point finite-difference approximation was used for the numerical calculation of the moment arm.

Based on the estimated musculotendon force and its corresponding moment arm, the net joint moment was calculated by adding the contributions of all relevant musculotendon units, as given in Equation (7). The obtained joint moment was then employed as the dynamic input for further lower-limb motion analysis.

**Figure 5:**
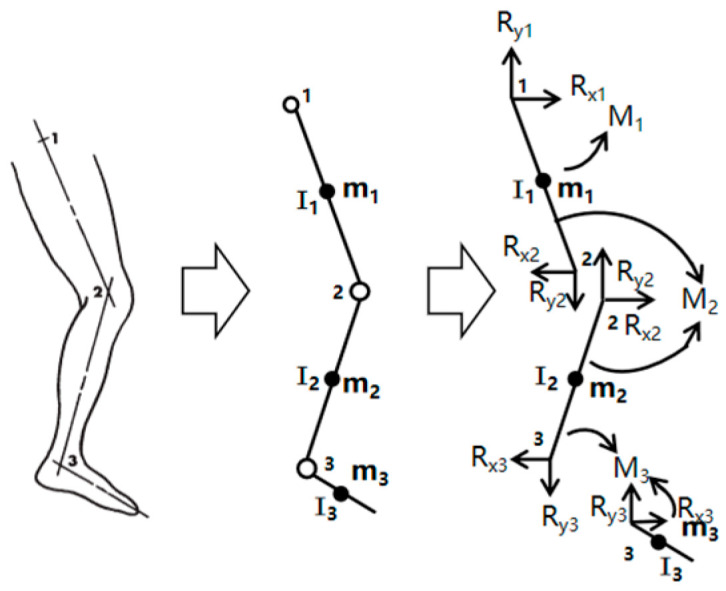
Model of lower limb rigid body dynamics.

To better understand human lower-limb kinematics, the lower extremity was represented as a rigid-link system. The rigid-body dynamic model of the lower limb is illustrated in Figure 5. Each body segment was identified by its mass, center of mass, and inertial properties, while joints were modeled using the anatomical kinematic structure. On this basis, the dynamic equations of the lower limb were established using the Newton–Euler method. The translational dynamics of each segment were described by Newton’s second law:

(11) $$F_{i}=m_{i}{\dot{v}}_{c,i}$$

where $F_{i}$ is the resultant external force acting on the *i*-th segment; $m_{i}$ is the segment mass; and ${\dot{v}}_{c,i}$ is the acceleration of its center of mass. The rotational dynamics were described by the Euler equation:

(12) $$T_{i}=I_{c,i}{\dot{w}}_{i}+w_{i}\times I_{c,i}w_{i}$$

where $T_{i}$ is the external moment acting on the *i*-th segment, $I_{c,i}$ is the inertia tensor about the center of mass, and $w_{i}$ is the angular velocity of the segment.

By combining the estimated joint moments with the segmental dynamic equations, the proposed framework was used to derive lower-limb dynamic outputs during gait, including knee joint motion and shank center-of-mass trajectory [41]. These variables were then utilized to evaluate the model, together with experimental reference data.

## 3. Experimental Results

### 3.1. Participants and Experimental Protocol

Gait experiments were conducted on stroke patients with hemiplegia to evaluate the proposed sEMG-driven musculoskeletal framework. Six subjects were recruited according to the requirements of the walking experiment, and their detailed characteristics are listed in Table 1. The inclusion criteria were as follows: each subject had unilateral hemiplegia caused by stroke, the contralateral lower limb remained functionally intact, the muscle strength of the affected lower limb had recovered to at least grade III after treatment, the subject was able to walk slowly and independently, and no other major physical impairments were present.

Each participant performed seven overground walking trials at a comfortable self-selected walking speed under a 20 beats/min metronome cue. Because hemiplegic participants may have difficulty maintaining a prescribed absolute walking speed or stride length, these variables were not forcibly standardized across subjects. Instead, the metronome was used to provide a consistent pacing cue while allowing safe and tolerable gait performance. During the experiment, a three-dimensional motion-capture system, a force measurement platform, and a surface electromyography system were used synchronously to record marker trajectories, ground reaction forces, and electromyographic amplitudes. Data acquisition started when the subject entered the measurement area and ended after the subject left the force platform and completed the entire walking task. A 15 min interval was provided between consecutive trials to minimize the influence of fatigue on the experimental data.

### 3.2. Model Evaluation Strategy

Because the knee joint exhibits substantial dynamic variation during gait, it was selected as the primary joint for biomechanical analysis and model validation. For each subject, model parameters were calibrated using the first five trials, and the remaining two trials were used to assess the model’s performance on previously unseen data from the same subject. This approach allows evaluation of the consistency of parameterized predictions within each individual. Model performance was evaluated by comparing biomechanical and motion variables estimated by the proposed framework with corresponding reference results obtained from inverse kinematics, forward motion analysis, and experimental measurements.

Three quantitative metrics were used for model evaluation: the correlation coefficient (R), the mean error (ME), and the root mean square error (RMSE). The correlation coefficient was used to assess the similarity in waveform trends between the estimated and reference signals, whereas ME and RMSE were used to quantify prediction bias and overall estimation error, respectively.

**Figure 6:**
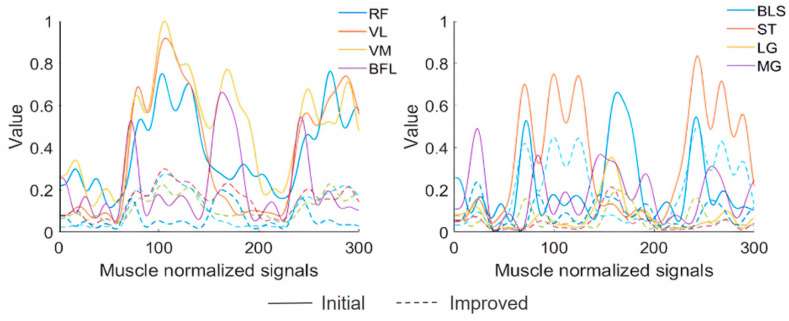
Comparison of initial and normalized muscle activation.

To better represent muscle activation in hemiplegic subjects, the normalized correction coefficient introduced in Section 2 was incorporated into the activation model, as shown in Figure 6. After the conventional normalized signal was obtained, an additional coefficient was assigned to each monitored muscle to improve the estimation of subject-specific activation levels. On this basis, a muscle activation dynamics model was adopted to estimate muscle activation, and the optimized results of representative model parameters for the six subjects are presented in Table 2.

### 3.3. Muscle Force Estimation

**Figure 7:**
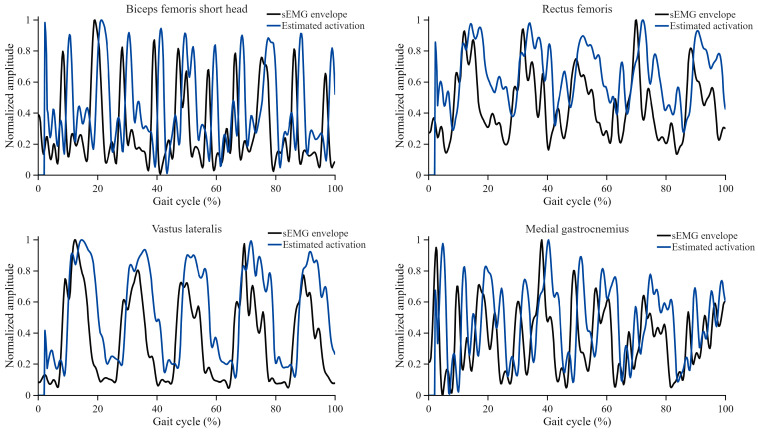
Comparison of recorded pathological sEMG envelopes and model-estimated activation profiles for subject 1.

To validate the reliability of the established muscle mechanics model, muscles with relatively high force-generating capacity were selected for analysis, including the short head of the biceps femoris, rectus femoris, vastus lateralis, and medial gastrocnemius. OpenSim static-optimization muscle forces were not interpreted as physiological ground truth for pathological muscle-force validation. Recorded pathological sEMG envelopes were compared with model-estimated activation profiles in Figure 7 to assess whether the model preserved the main activation burst patterns observed experimentally. This evaluation focuses on the consistency of activation timing, including onset, offset, and main burst patterns, rather than absolute muscle-force magnitudes. The agreement in burst timing indicates that the activation model preserved the main temporal characteristics of the recorded pathological muscle activity.

The predicted muscle-force profiles showed similar temporal patterns to the reference simulations. The estimated muscle-force curves reproduced the major temporal characteristics of muscle-force generation during gait, indicating that the proposed model can effectively capture the main variation trends of the selected muscles during the gait cycle.

### 3.4. Knee Joint Torque Estimation

**Figure 8:**
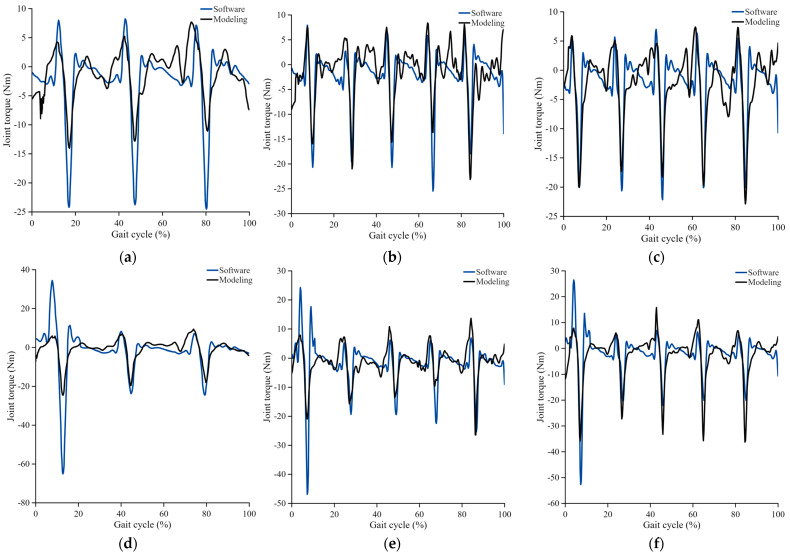
Calculation and simulation results: (**a**) subject 1 result; (**b**) subject 2 result; (**c**) subject 3 result; (**d**) subject 4 result; (**e**) subject 5 result; (**f**) subject 6 result.

Using the calibrated activation dynamics and the improved Hill-type muscle model, the knee joint torque of the affected limb was estimated for all six subjects over one complete gait cycle. The validation indices for the six subjects, including correlation coefficient (R), mean error (ME), root-mean-square error (RMSE), RMSE normalized to body mass (Nm/kg), and RMSE expressed as a percentage of peak knee joint torque (nRMSE), are summarized in Table 3, and the corresponding calculated and simulated results are shown in Figure 8.

Overall, the calculated knee joint torque curves were consistent with the software simulation results for all six subjects, indicating that the established model can estimate knee joint torque with good reliability. The predicted torque profiles reproduced the more temporal variation patterns observed in the reference results during hemiplegic gait.

### 3.5. Knee Joint Motion and Shank Center-of-Mass Trajectory Results

**Figure 9:**
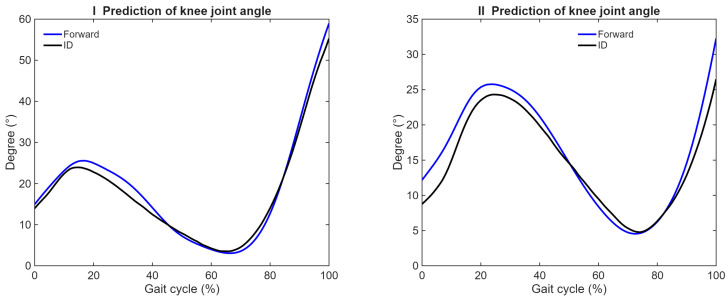
Estimation of the knee joint angle.

**Figure 10:**
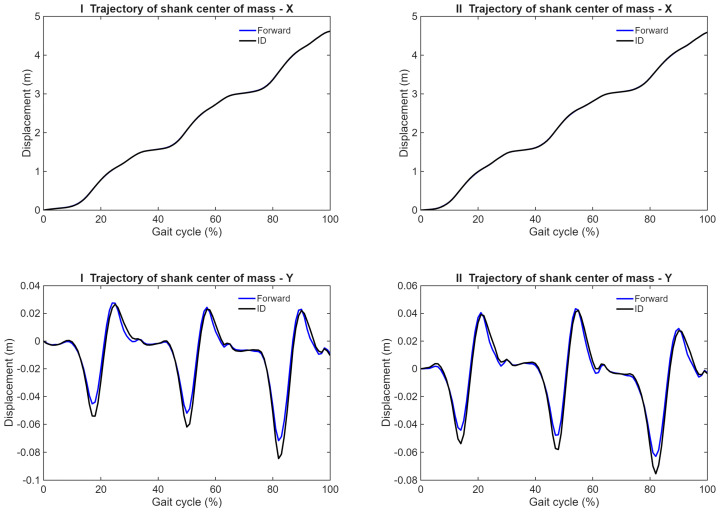
Estimation of the trajectory of the center of mass.

Based on the estimated joint torque, the mapped joint torque output was further input into the forward joint motion model of the human body to calculate the knee joint motion angle of the lower limb, as shown in Figure 9. In addition, the shank center-of-mass trajectory was calculated and expressed relative to the initial contact frame to avoid dependence on arbitrary global laboratory coordinates. As illustrated in Figure 10, each trajectory was zeroed by subtracting its first sample from all subsequent samples in both the x- and y-directions. Thus, the resulting curves represent the displacement of the shank center of mass relative to initial contact.

All estimated motion variables were further compared with the corresponding reference measurements. For the shank center-of-mass variables, the comparison was performed using the initial-contact-zeroed displacement trajectories described above. The correlation coefficients, RMSE values, and ME values between the reference measurements and model predictions are summarized in Table 4. These results indicate that the proposed framework reproduced the main joint-level kinematic characteristics and segment-level displacement patterns during hemiplegic gait.

## 4. Discussion

OpenSim static-optimization muscle forces are not interpreted as ground-truth pathological muscle forces. The analysis focuses on the consistency of activation timing between the model-estimated activations and recorded pathological sEMG envelopes. Minor discrepancies in specific muscles, such as the rectus femoris, vastus lateralis, and biceps femoris short head, are expected due to algorithmic formulation, parameter scaling, and software-specific optimization strategies. This approach emphasizes qualitative agreement and preservation of activation timing rather than direct quantitative validation of pathological muscle forces.

The estimated knee joint torques showed moderate to good agreement with inverse-dynamics-derived references on held-out trials from the same subjects, providing computational validation. These results should be interpreted as computational agreement rather than independent physiological validation. Independent measurements, such as dynamometer-based torque, ultrasound-informed muscle fascicle behavior, instrumented gait benchmarks, and prospective clinical correlations, are recommended for future validation. The personalized model established in this study also showed good performance in knee joint torque estimation and provided biomechanical information for subject-specific analysis in hemiplegic gait. However, certain systematic errors remained. These errors may mainly arise from two sources. First, abnormal gait patterns in hemiplegic subjects can introduce deviations in marker trajectories and ground reaction force measurements, thereby affecting the accuracy of inverse-dynamics calculations. Second, neuromuscular control impairment often leads to irregular electromyographic activity and reduced signal quality, which may further affect the estimation of muscle activation and force output. The revised parameter constraints reduce the risk of parameter equivalence, but do not completely eliminate parameter-identifiability challenges inherent to sEMG-driven musculoskeletal modeling. Future work should incorporate independent physiological measurements, ablation studies, and formal sensitivity analyses to further constrain and interpret the parameter space.

From a clinical perspective, the estimated biomechanical patterns can be interpreted in the context of known mechanisms of hemiplegic gait. Reduced or delayed knee-extension moments may reflect insufficient quadriceps contribution during stance, while abnormal timing or diminished activation of the gastrocnemius muscles may indicate impaired plantarflexor function and reduced forward propulsion. Overlapping activation between knee extensors and flexors likely reflects compensatory co-contraction, a common feature of pathological gait that can contribute to stiff-knee patterns. Descriptive observations also suggest that subjects with higher Brunnstrom stages or longer recovery durations tend to exhibit better waveform agreement. These findings are preliminary and should not be interpreted as statistically confirmed clinical associations. Future studies with larger cohorts and standardized clinical scales, including FMA, MAS, FAC, Brunnstrom stage, and walking ability, are warranted to further explore these relationships.

Inter-subject differences in model performance were evident across all six participants. Subjects 1 and 3 demonstrated relatively higher agreement with inverse-dynamics-derived joint moments, while Subjects 2, 4, 5, and 6 showed comparatively lower agreement, as indicated by correlation R, RMSE, RMSE normalized to body mass, and nRMSE relative to peak joint torque. These variations likely reflect clinical heterogeneity among participants, including differences in rehabilitation duration, Brunnstrom stage, and neuromuscular recovery. Overall, these observations highlight that subject-specific clinical characteristics can substantially influence model performance and should be taken into account when interpreting results and designing future studies with larger and more clinically diverse cohorts.

The main potential value of the proposed framework lies in its ability to estimate subject-specific biomechanical characteristics using a limited set of clinically collectible gait and sEMG data, which is consistent with the need for quantitative assessment methods in clinical motor-function evaluation [42]. For hemiplegic patients, conventional MVC-based normalization is often difficult or unsafe to perform, and repeated physical testing may increase fatigue or the risk of secondary injury. Under such conditions, the present framework may provide a non-invasive means for subject-specific biomechanical assessment and may support the design of individualized rehabilitation strategies. In addition, differences between the estimated muscle-force profiles and the generic simulation results may help reveal altered muscle function during gait, as similar biomechanical comparisons have been used in previous studies to interpret neuromuscular performance [43]. For example, some muscles in specific subjects showed force patterns that differed from the generic baseline across gait phases, which may reflect abnormal or compensatory neuromuscular behavior. However, such interpretations should be made cautiously and require further validation with additional clinical evidence.

Another noteworthy finding is that the proposed framework did not stop at joint torque estimation, but further propagated the estimated torque through the lower-limb dynamic model to recover knee joint motion and shank center-of-mass trajectory. This extension is meaningful because previous studies have primarily focused on torque estimation under relatively constrained conditions, with less attention given to dynamic motion outputs during functional daily activities [44]. In this sense, the present work extends previous studies that primarily focused on torque estimation under relatively constrained conditions and demonstrates the feasibility of linking muscle-force estimation to subject-specific dynamic motion outputs.

Despite these encouraging results, several limitations should be acknowledged. First, although the number of participants was increased in the revised study, the sample size remained limited, which restricts the generalizability of the present findings. Second, model validation focused mainly on knee joint torque and selected muscle-level activation characteristics, without systematic evaluation of hip and ankle biomechanics. Third, cross-subject validation was not performed, and the robustness of the optimized parameters therefore remains uncertain. Small, heterogeneous clinical gait datasets present significant challenges in rehabilitation research. Data collection from hemiplegic patients is often limited by recruitment constraints, patient fatigue, safety considerations, and the complexity of simultaneously acquiring motion capture, force plate, and sEMG signals [26]. Although the proposed framework is mechanistic rather than data-driven, it faces a similar challenge: extracting reliable biomechanical insights from limited, heterogeneous data. Recent work by Trabassi et al. on rare disease gait classification using data balancing and generative AI underscores the importance of handling small and imbalanced datasets to improve interpretability. Future integration of mechanistic modeling with data-driven strategies, such as data augmentation, Bayesian parameter estimation, and physics-informed learning, may enhance robustness and generalizability while preserving biomechanical interpretability. Although $F_{max}$ was fixed to reduce equifinality with the sEMG correction coefficient *c*, residual parameter-identifiability limitations may still exist because the model remains calibrated using subject-specific biomechanical data. In addition, the optimized correction coefficient c should not be interpreted as a direct clinical measure of spasticity or voluntary activation capacity. The geometric assumption used for dynamic pennation-angle estimation may also not fully capture architectural changes in hemiplegic muscles. Finally, the mapped joint torque still relies on inverse-dynamics data, which limits the immediate applicability of the framework in fully predictive or real-time assistive scenarios. Future studies should expand sample size, include subjects with more diverse clinical characteristics, incorporate independent measurements of muscle architecture, and further develop a more predictive forward-dynamics module to improve the generalizability and practical applicability of the model.

## 5. Conclusions

In this study, a subject-specific lower-limb musculoskeletal modeling framework for hemiplegic patients was developed. Based on anatomical analysis and patient-specific marker data, a personalized musculoskeletal model was established. By incorporating a normalized correction coefficient and submaximal activation conditions, the proposed framework enabled reliable mapping from sEMG signals to muscle activation without relying on conventional MVC testing. On this basis, an improved Hill-type muscle model and parameter-optimization procedure were used to estimate musculotendon force, knee joint torque, knee joint kinematics, and the shank center-of-mass trajectory. The overall results showed moderate to good agreement with the corresponding reference data, supporting the feasibility of the proposed framework for subject-specific biomechanical analysis in hemiplegic gait.

In summary, the proposed framework provides preliminary proof-of-concept evidence for subject-specific biomechanical estimation in hemiplegic gait. It may serve as an interpretable and non-invasive computational approach for individualized rehabilitation assessment. Given the limited cohort size, future studies with larger and more clinically diverse samples, cross-subject validation, and independent physiological measurements are needed to further evaluate its robustness and clinical applicability.

## Figures and Tables

**Table 1:** Experimental personnel information.

Subject	Gender/Age	Height/Weight	Hemiplegic Side	Course	Brunnstrom
1	male/58	175/72	right	five months	V
2	male/53	178/78	right	three months	IV
3	male/49	172/65	right	four months	V
4	male/60	168/63	right	three months	IV
5	female/55	165/60	left	six months	V
6	male/48	178/75	left	three months	IV
Mean ± SD	-/53.8 ± 4.8	172.7 ± 5.4/68.8 ± 7.2	-	-	-

**Table 2:** Partial model optimization parameters for subjects.

Optimization Parameters	Muscles	Initial Value	Subject 1	Subject 2	Subject 3	Subject 4	Subject 5	Subject 6
sEMG correction coefficient c	RF	0.50	0.10	0.37	0.56	0.34	0.10	0.11
	VL	0.50	0.10	0.99	0.99	0.96	0.10	0.10
	VM	0.50	0.10	0.10	0.32	0.16	0.10	0.27
	BFL	0.50	0.10	0.99	0.99	0.99	0.10	0.10
	BFS	0.50	0.16	0.10	0.12	0.14	0.13	0.17
	ST	0.50	0.47	0.99	0.58	0.92	0.28	0.21
	LG	0.50	1.00	0.98	0.96	0.99	0.75	1.00
	MG	0.50	0.10	0.68	0.43	0.68	0.10	0.10
Damping Coefficients B	RF	0.50	1.00	0.32	0.53	0.62	1.00	1.00
	VL	0.50	0.01	0.08	0.78	0.85	0.01	0.01
	VM	0.50	0.05	0.03	0.02	0.05	0.07	0.01
	BFL	0.50	1.00	0.85	1.00	0.86	0.98	1.00
	BFS	0.50	1.00	0.30	0.04	0.23	0.06	0.01
	ST	0.50	0.95	0.95	0.96	0.98	0.98	1.00
	LG	0.50	1.00	0.40	0.06	0.32	0.05	0.01
	MG	0.50	0.01	0.22	0.01	0.32	0.32	0.01
sEMG Delay d	/	10.00	20.00	20.00	20.00	20.00	20.00	20.00
Neural Activation Factors	/	0.50	0.66	0.43	0.35	0.62	0.50	0.66
		0.50	0.48	0.41	0.49	0.43	0.45	0.48
Muscle Activation Factors	/	−1.50	−2.31	−2.66	−2.45	−2.56	−2.34	−2.31
Percentage Change Factors	/	0.15	0.25	0.23	0.24	0.23	0.23	0.25

**Table 3:** Model prediction result indicators.

Subject	R	ME (Nm)	RMSE (Nm)	RMSE/Body Mass (Nm/kg)	nRMSE (% Peak Torque)
1	0.724	3.104	4.013	0.056	18.112
2	0.742	3.061	4.151	0.058	16.936
3	0.738	2.920	3.872	0.054	15.203
4	0.747	3.071	3.924	0.055	17.709
5	0.740	3.848	5.550	0.077	10.531
6	0.807	4.366	7.814	0.109	12.002

**Table 4:** Measurement index.

Index	Correlation	Mean Error	Root Mean Square
Data			
Knee Angle	0.99	1.541°	1.938°
Shank Position-X	0.99	0.002 m	0.003 m
Shank Position-Y	0.99	0.003 m	0.005 m

## Data Availability

The data and code for the current study are available from the corresponding author upon reasonable request.
